# Cell-Based Reporter Release Assay to Determine the Potency of Proteolytic Bacterial Neurotoxins

**DOI:** 10.3390/toxins10090360

**Published:** 2018-09-05

**Authors:** Andrea Pathe-Neuschäfer-Rube, Frank Neuschäfer-Rube, Gerald Haas, Nina Langoth-Fehringer, Gerhard Paul Püschel

**Affiliations:** 1 Department of Nutritional Biochemistry, Institute of Nutritional Science, University of Potsdam; Arthur-Scheunert-Allee 114-116, 14558 Nuthetal, Germany; fneusch@uni-potsdam.de (F.N.-R.); gpuesche@uni-potsdam.de (G.P.P.); 2 Croma-Pharma GmbH, Cromazeile 2, A-2100 Leobendorf, Austria; gerald.haas@croma.at (G.H.); langoth-fehringer@croma.at (N.L.-F.)

**Keywords:** botulinum toxin, BoNT, tetanus toxin, RRR, replacement

## Abstract

Despite the implementation of cell-based replacement methods, the mouse lethality assay is still frequently used to determine the activity of botulinum toxin (BoNT) for medical use. One explanation is that due to the use of neoepitope-specific antibodies to detect the cleaved BoNT substrate, the currently devised assays can detect only one specific serotype of the toxin. Recently, we developed a cell-based functional assay, in which BoNT activity is determined by inhibiting the release of a reporter enzyme that is liberated concomitantly with the neurotransmitter from neurosecretory vesicles. In theory, this assay should be suitable to detect the activity of any BoNT serotype. Consistent with this assumption, the current study shows that the stimulus-dependent release of a luciferase from a differentiated human neuroblastoma-based reporter cell line (SIMA-hPOMC1-26-GLuc cells) was inhibited by BoNT-A and-C. Furthermore, this was also inhibited by BoNT-B and tetanus toxin to a lesser extent and at higher concentrations. In order to provide support for the suitability of this technique in practical applications, a dose–response curve obtained with a pharmaceutical preparation of BoNT-A closely mirrored the activity determined in the mouse lethality assay. In summary, the newly established cell-based assay may represent a versatile and specific alternative to the mouse lethality assay and other currently established cell-based assays.

## 1. Introduction

Botulinum toxin (BoNT) is one of the most potent neurotoxins known, which has a lethal dose of 1–3 ng/kg body weight in human adults. The exotoxin is produced by the obligatory anaerobic bacteria, which are namely Clostridium botulinum, Clostridium butyricum and Clostridium baratii [1,2]. Currently, at least seven serotypes and several subtypes of the toxin are known [3]. Accidental intoxication, which is known as botulism, results from the ingestion of insufficiently heated preserved food or systemic production of the toxin after the germination of spores in wounds or in the immature intestine of infants [4]. The toxin causes a sustained inhibition of the release of acetyl choline from the motor endplate, resulting in flaccid paralysis. Death occurs from asphyxia. Despite intensive care medical interventions, the overall lethality of botulism is still around 3%.

The long lasting blockage of neurotransmitter release from nerve endings is exploited in the treatment of a number of neurological ailments, such as torticollis spasmodicus and strabismus, or non-neurological pathological conditions, such as hyperhidrosis or urinary bladder dysfunction. However, in recent years, the main field of medical use of BoNT is in aesthetic medicine, in which the toxin is used to remove wrinkles by locally paralyzing cutaneous muscles [5,6,7]. Due to its extreme toxicity, every batch of the pharmacological preparations of BoNT must be vigorously tested to determine its content of active toxin. A mouse lethality assay has long been the gold standard and still is widely used [8].

The bacterial toxin is composed of a large and a small subunit that are connected by a disulfide bond. The toxin is secreted in a complex with additional proteins that play a role in its intestinal absorption [9]. Once in the circulation, the large subunit of the toxin binds to proteins and carbohydrate receptor structures on the surface of neuronal cells and is taken up by endocytosis [10]. In the acidic environment of the endocytotic vesicle, the small subunit is detached from the large subunit. After this, the large subunit catalyzes the transport of the small subunit from the vesicle into the cytosol of the nerve cell. Here, the small subunit encounters the substrate molecules for its proteolytic activity, which are the proteins involved in the fusion of the neurosecretory vesicle with the presynaptic plasma membrane (SNARE proteins) [11]. Cleavage of these substrate proteins blocks the vesicle fusion with the plasma membrane and hence, neurotransmitter release. Different BoNT serotypes use different receptor structures to enter the cells and cleave different target proteins. Thus, BoNT-A binds to Synaptic vesicle protein 2 C/A/B (SV2C/A/B) and cleaves Synaptosome-associated protein 25 kDa (SNAP25), while BoNT-B binds to Synaptotagmin I/II (Syt-II/Syt-I) and cleaves Vesicle-associated membrane proteins (VAMP). The protein receptor for BoNT-C, which cleaves SNAP25 and Synthaxin, is unknown [10,11].

Due to the ethical concerns that are inherent in the mouse lethality assay, alternative methods to determine BoNT activity are currently needed. Activity testing must determine both the interaction with the receptor structures and the cleavage of the substrate proteins. Most in vitro assays can determine only one of these steps [12]. However, several in vitro and cell-based assays have been recently described that measure both the interaction of the large subunit with the receptor structures and the enzymatic activity of the small subunit. For example, the BINACLE test first captures the toxin in test plates, taking advantage of the interaction of the large subunit with immobilized receptor structures. In a second step, the large and small subunits are separated by reduction, before the activity of the small subunit in the supernatant is determined by the immunological quantification of neo-epitopes generated on an immobilized substrate in a second well [13]. A similar approach is taken in cell-based assays, in which the BoNT-A is taken up into the cells of a differentiated human neuronal cell line (SIMA cells) [14] or neuronal cells derived from hiPSC [15] and the cleavage of SNAP25 is quantified by a SNAP25 cleavage product-specific ELISA. Both systems suffer from a major limitation as they only work for one specific BoNT subtype.

Recently, we developed a cell-based assay that determines the BoNT activity by quantifying the inhibition of the release of a luciferase, which has been redirected into neurosecretory vesicles. This can act as a surrogate for the inhibition of neurotransmitter release [16]. In theory, this assay should overcome the limitations of the other currently established in vitro or cell-based assays as it should be suitable for all BoNT serotypes and for the tetanus toxin, which also cleaves the VAMP protein. Therefore, the aim of the current study was to provide a proof of principle that the test is suitable for different serotypes and can be used to determine the biological activity of botulinum toxin in the pharmaceutical preparations of BoNT. 

## 2. Results

### 2.1. Suitability for Testing BoNT Activity in Pharmacological Preparations

**Figure 1:**
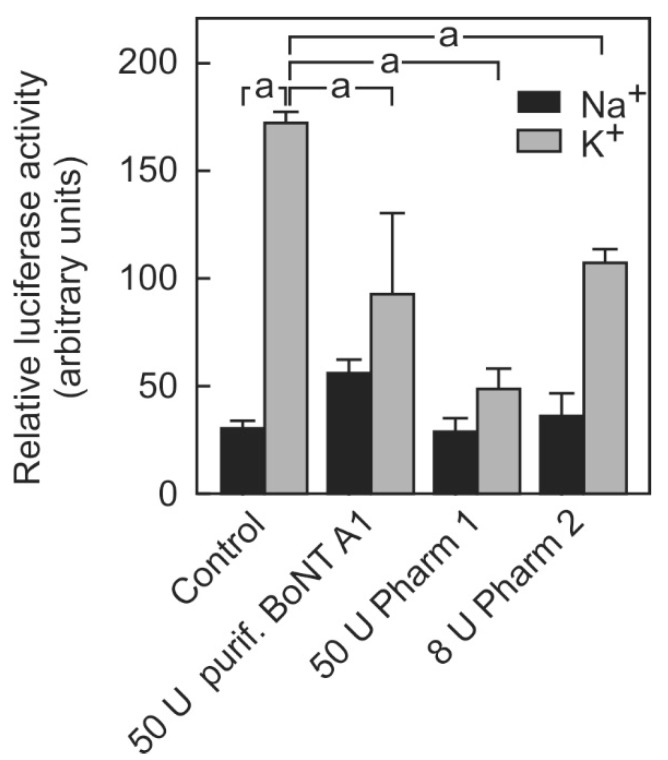
Inhibition of stimulation-dependent luciferase release by purified BoNT-A and pharmacological BoNT-A preparations. SIMA cells stably expressing hPOMC1-26-GLuc were cultured and differentiated as described in the methods section. They were incubated with the indicated mouse lethality units of either purified botulinum toxin A1 or pharmacological botulinum toxin preparations by two different providers (Pharm 1 and Pharm 2) for 48 h. After removing the remaining toxin from the supernatant and a brief recovery phase (see methods section), cells were incubated for three minutes with non-depolarizing (Na^+^) or depolarizing (K^+^) balanced salt solutions. Cell culture supernatants were centrifuged and luciferase activity was determined in the cell culture supernatants. Values are means ± SEM of 2–3 independent experiments. Statistics: Student’s t-test for unpaired samples, a: *p* < 0.05.

To assess the suitability of the luciferase release assay for testing the BoNT activity in pharmacological preparations, the toxins of two providers were tested in comparison with purified BoNT A (Figure 1). 

When the cells were incubated with a non-depolarizing Na^+^-containing buffer, a certain amount of luciferase activity occurred in the cell culture supernatant. This unspecific release was not affected by botulinum toxin treatment. When the cells were stimulated with a K^+^-containing depolarizing buffer, the amount of luciferase released into the medium was approximately 4-fold higher than the unspecific release. The specific release was inhibited by about 50% after incubation with 50 mouse lethality units (MLU) of a purified BoNT-A. A total of 50 MLU of a BoNT-A1 preparation (Pharm 1) inhibited the luciferase release to a similar extent. Although 8 MLU units of BoNT-A1 preparation (Pharm 1) no longer significantly inhibited the release of luciferase under these not yet optimized conditions (not shown), 8 U of a BoNT A1 preparation (Pharm 2) caused a significant inhibition of 25% in the depolarization-stimulated luciferase release. Higher concentrations were not tested due to the limited availability of the drug product. To exclude any possibility that the additives contained in the pharmaceutical preparations of BoNT might contribute to the inhibition of the reporter release, BoNT-A1 (Pharm 1) was heat-inactivated prior to the incubation of the cells. Heat-inactivated BoNT-A1 (Pharm 1) no longer affected the stimulus-dependent reporter release (data not shown). Thus, the cell-based assay was suitable to determine the activity of BoNT-A in pharmacological preparations although the mouse lethality units provided by the manufacturers appeared not to directly reflect the activity of BoNT in this assay.

The assay conditions were optimized to increase the sensitivity and reduce the amount of toxin needed for activity determination. This included a reduction in the incubation volume and the number of cells used per assay point.

**Figure 2:**
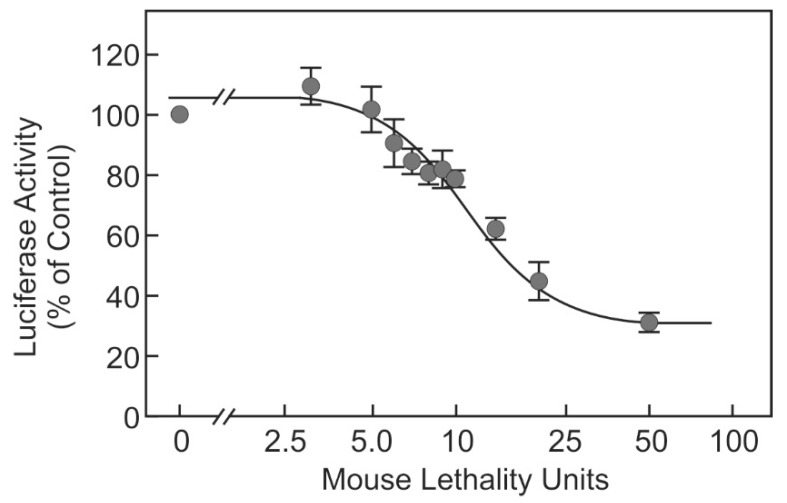
Dose-dependent inhibition of luciferase release by pharmaceutical BoNT-A1. SIMA cells stably expressing hPOMC1-26-GLuc were cultured and differentiated as described in the methods section. Cells were incubated with the indicated mouse lethality doses of BoNT-A1 (Pharm 1). Stimulus-dependent release of luciferase activity was determined in the cell culture supernatants. The luciferase activity by cells stimulated without prior BoNT incubation was set as 100% in each experiment. Values are means ± SEM of at least nine independent experiments.

A dose–response curve was generated using the BoNT-A1 (Pharm 1) (Figure 2). BoNT-A1 (Pharm 1) inhibited the stimulus-dependent luciferase release dose-dependently in a concentration range of 5–25 MLU. The lowest concentration that resulted in a statistically significant inhibition was 7 MLU. In the concentration range of 5–20 MLU, the steps of at least 4–5 MLU caused significant differences in the extent of the inhibition of luciferase release.

### 2.2. Suitability of the Assay for Different BoNT Serotypes

**Figure 3:**
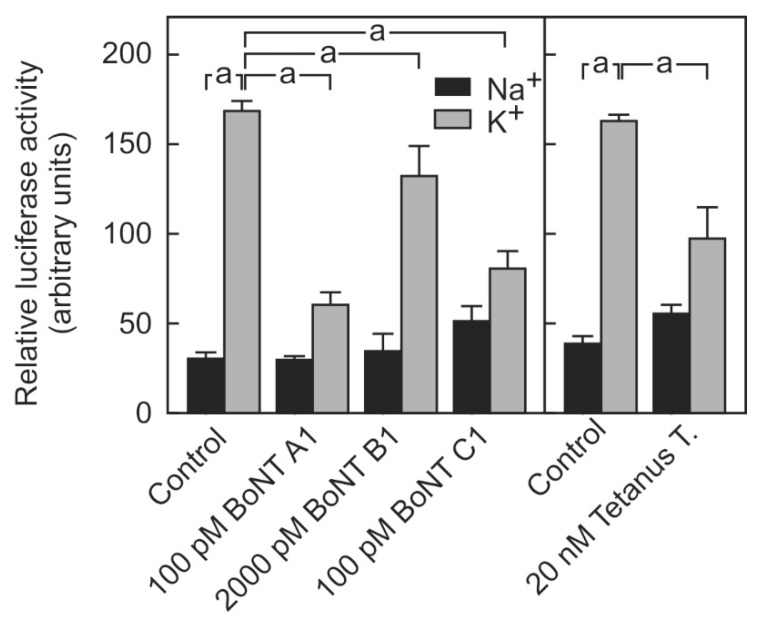
Inhibition of stimulation-dependent luciferase release by purified BoNT-A, BoNT-B, BoNT-C and Tetanus toxin. SIMA cells stably expressing hPOMC1-26-GLuc were cultured and differentiated as described in the methods section. Cells were incubated with the indicated concentration of the respective neurotoxin and luciferase release was determined as described in legend to Figure 1. Values are means ± SEM of 5–7 independent experiments. Statistics: Student’s t-test for unpaired samples, a: *p* < 0.05.

To test the hypothesis that the reporter system is suitable for determining the activity of different BoNT serotypes, the cells were exposed to purified BoNT-A, purified BoNT-B and complex BoNT-C. Both 100 pM BoNT-A, which corresponds to 211 MLU according to the manufacturer, and 100 pM BoNT-C, which corresponds to 76 MLU, caused a similar inhibition of 66% in the stimulus-dependent luciferase release (Figure 3). This is equivalent to the maximally achieved inhibition of stimulus-dependent luciferase release.

By contrast, 100 pM BoNT-B, which corresponds to 65 MLU, did not inhibit the stimulus-dependent luciferase release (not shown). BoNT-B could only significantly inhibit the stimulus-dependent luciferase release at 20-fold higher concentrations (2000 pM, which corresponds to 1300 MLU) although this amounted to only roughly 30%. BoNT-B shares the same substrate with tetanus toxin, i.e., VAMP. Therefore, the impact of tetanus toxin on the stimulus-dependent luciferase release was tested (Figure 3). Significantly higher concentrations of the tetanus toxin were needed to inhibit luciferase release. At a concentration of 20 nM, the toxin inhibited the depolarization-dependent luciferase release by about 50% without significantly affecting the non-specific luciferase release after incubation with a non-depolarizing sodium-containing buffer (Figure 3). 

**Figure 4:**
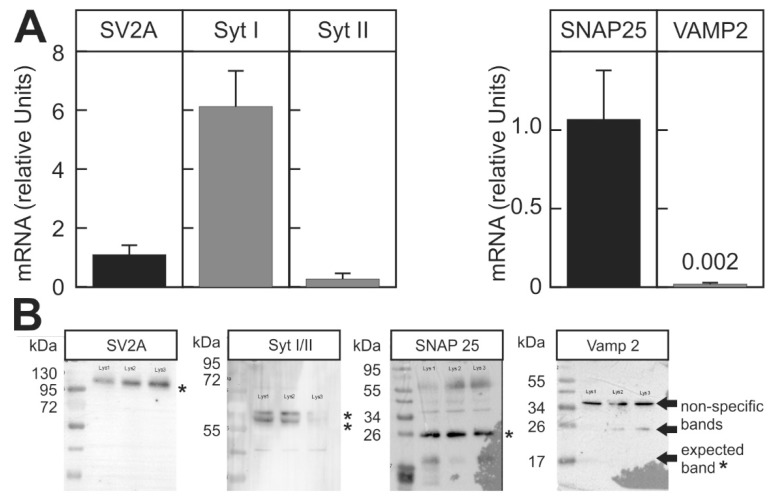
Expression of receptor and substrate proteins for the different BoNT serotypes in the reporter cell line. The expression of the receptor and substrate proteins for the different BoNT serotypes was determined in differentiated SIMA cells stably expressing hPOMC1-26-GLuc. (**A**) mRNA was isolated and relative mRNA expression was determined by RT-qPCR, as described in the methods section. Expression level of SV2A and SNAP25 was arbitrarily set as 1 for the comparison of the mRNA of the receptor and substrate proteins, respectively. Values are means ± SEM of 3 independent mRNA preparations. (**B**) Protein expression of the different proteins was determined in three independently produced cell lysates (indicated by the non-readable text on the blot membranes) with specific antibodies by Western blot. Asterisks indicate the expected specific bands.

BoNT requires the protein receptor structures to enter the nerve cells. Therefore, the strong inhibition of stimulus-dependent luciferase activity by BoNT-A and BoNT-C and the weak inhibition of stimulus-dependent luciferase release by BoNT-B might be due to the differences in the expression of the respective receptor proteins. However, this hypothesis was not supported as we found that SV2A, the receptor for BoNT-A, and Syt I, the receptor for BoNT-B, were expressed at comparable levels in the reporter cell line both at the mRNA and protein levels (Figure 4). Alternatively, the low sensitivity towards BoNT-B might be caused by excessively high levels of the substrate. However, qPCR and protein data indicate that the expression of the BoNT B substrate VAMP-2 was actually lower than the expression of the SNAP25, the substrate for the small subunit of BoNT-A and C. This finding was confirmed by Western blot, which found that VAMP-2 in contrast to SNAP25 was not detectable (Figure 4B).

## 3. Discussion

### 3.1. Suitability of the Sssay as Replacement Method

Here, we show that our recently developed cell-based neurotransmitter release reporter assay was suitable to determine the activity of pharmacological BoNT preparations and hence, might be suitable as a replacement method for the mouse lethality assay that currently is still frequently used to determine the activity of the enzyme. The stimulus-dependent reporter release was inhibited by two different pharmaceutical preparations of BoNT-A1 (Pharm 1 and Pharm 2) (Figure 1). The extent of inhibition of luciferase release by BoNT was stable over several passages of the cell line (not shown). The reporter release was dose-dependently inhibited by BoNT-A1 (Pharm 1 in a concentration range that corresponds to 5–25 MLU, i.e., the content of 1/10 to 1/2 of a vial in a ready to use batch) (Figure 2).

Recently, two other cell-based assays have been described, which are used as a replacement method for the mouse lethality assay [14,17]. Although the former assay uses SIMA cells, such as the assay described here, the latter uses differentiated neurons derived from induced pluripotent stem cells. Although both assays determine the activity of the small and the large subunits of BoNT-A, they suffer from a major limitation. Both assays depend on the detection of a neo-epitope generated by the cleavage of SNAP25 by the small subunit of BoNT-A and hence, are strictly specific for the serotype of toxin they were designed for. A major advantage compared to the current assay described in this study is the apparent greater sensitivity (see below). 

A different approach to test the functionality of both large and small subunits of BoNT-A is followed by the BINACLE assay [13]. Here, the toxin is first captured by the interaction of the large subunit with immobilized receptor proteins, which is followed by the detection of a neo-epitope generated on an added substrate. The major advantage of this assay is that it is a completely cell-free assay, which: (1) avoids the comparatively high infrastructural requirements of eukaryotic cell culture and (2) can be more readily standardized because all assay components of this in vitro system are well characterized. However, this detection method is suitable only for one specific serotype of botulinum toxin. 

The current assay overcomes this problem. The reporter enzyme release was also inhibited by BoNT-C and at higher concentrations, by BoNT-B and tetanus toxin (Figure 3). These results demonstrate that the newly developed assay is appropriate to determine the activity of botulinum toxin independently of the serotype and may also be used to determine the activity of different neurotoxins, whose small subunit cleaves SNARE proteins involved in neurotransmitter release. 

A possible future improvement of the current assay involves the use of a cell impermeable luciferase substrate, which has been described by Takakura et al. [18]. This would allow the determination of the stimulus-dependent luciferase release in real time directly using the cell culture plate as a continuous one step assay since only the released luciferase would interact with the substrate. The sampling and centrifugation of the cell culture supernatant after the stimulation of the release, which is currently necessary, would be dispensable. 

A potential disadvantage of the current assay is its limited sensitivity. Both cell-based assays detecting the BoNT-dependent formation of a neo-epitope and the BINACLE assay detect botulinum toxins in concentrations that correspond to less than one MLU and hence, are about ten-fold more sensitive.

### 3.2. Potential Reasons for the Limited Sensitivity for BoNT-B

Although BoNT-A and BoNT-C inhibited the stimulus-dependent luciferase release at similar low concentrations, BoNT-B failed to inhibit the stimulus-dependent luciferase release at low concentrations and BoNT-B at the maximum feasible concentration tested (2 nM) only caused a 30% inhibition (Figure 3). The possible explanations could be that the receptor structures needed for the uptake of the toxin are expressed in the reporter cell line at low levels or not at all or that the substrate SNARE protein is expressed at exceedingly high concentrations, overwhelming the capacity of the small subunit for proteolytic cleavage. However, both hypotheses were refuted. First, the putative receptor protein for BoNT-B is Syt II. Furthermore, the receptor was detected on the reporter cells both on the mRNA and protein levels (Figure 4). However, human Syt II apparently has a more than 100-fold lower affinity for BoNT-B than the mouse homolog [19], providing a possible reason why BoNT-B was less effective in the cell based assay using a human neuronal cell line. If this was the reason, this newly developed cell-based assay would actually be more relevant to evaluate the potency of a toxin that is meant to be used in human therapy as it is not confounded by inter-species differences in sensitivity. Notably, 66-fold higher doses of BoNT-B than of BoNT-A (10000 MLU vs. 150 MLU) were needed to achieve similar results in the treatment of cervical dystonia in patients who had previously not been treated with either toxin (toxin-naive patients) [20]. 

Surprisingly, the expression of the substrate of BoNT-B was very low on the mRNA level and not detectable by the immunoblot on the protein level (Figure 4). The low expression level of VAMP-2 in SIMA cells and other neuroblastoma cell lines, such as SHSY-5Y, was shown independently by others [21]. In contrast to the determination of neo-epitope-dependent activity, which requires high concentrations of the substrate proteins in the test cell line, necessitating transgenic expression of the protein to improve sensitivity of the assay [21], the low expression of the substrate SNARE protein in the current functional assay would correspond to high sensitivity since the functional reserve of SNARE protein left after partial cleavage by the enzyme would be low. Although VAMP-2 was not detectable by Western Blot, its presence in SIMA cells, albeit at very low concentrations, was confirmed by the inhibition of stimulus-dependent luciferase release by the incubation of the cells with tetanus toxin (Figure 3). The small subunit of tetanus toxin also specifically cleaves VAMP-2 at the same site as BoNT-B [22].

## 4. Conclusions

In conclusion, the newly established cell-based assay may represent a versatile alternative to the mouse lethality assay and currently established cell-based assays with a narrow serotype specificity. In addition, using a human reporter cell line avoids inter-species biases and therefore, this assay will provide more accurate patient-relevant activity information than the mouse lethality assay.

## 5. Materials and Methods 

### 5.1. Materials 

All chemicals were purchased from commercial sources indicated throughout the text. Oligonucleotides were custom-synthesized by Eurofins Operon (Ebersberg, Germany) or Biolegio (Nijmegen, The Netherlands). The antibodies used were SNAP25 #5309, Cell Signaling, Frankfurt am Main, Germany; GAPDH sc-25778, VAMP-1/2/3 sc-133129, SytI/II sc-393392 and SV2A sc-376234, Santa Cruz Biotechnology, Heidelberg, Germany). 

### 5.2. Cell Culture

The generation of the stably transfected human neuroblastoma cell line SIMA hPOMC1-26-GLuc has been described previously [16]. Cells were cultured in RPMI 1640 medium (Biochrom, Berlin, Germany) supplemented with 10% (*v*/*v*) heat-inactivated fetal calf serum (FCS, Biochrom, Berlin, Germany), while 2 mM stable l-alanyl-l-glutamine and penicillin (100 U/mL)/streptomycine (100 µg/mL) (Biochrom, Berlin, Germany) were used as antibiotics.

### 5.3. Luciferase Release from BoNT or TeT Treated Cells 

For luciferase release experiments, SIMA-hPOMC1-26-GLuc cells were differentiated in poly-l-lysine-coated 96-well plates (5 × 10^3^–5 × 10^4^ cells/well) with a differentiation medium (RPMI supplemented with 1 × B27 supplement, 1 × N2 supplement (Thermo Scientific, Darmstadt, Germany, 2 mM l-alanyl-l-glutamine, 1 mM non-essential amino-acids (Biochrom, Berlin, Germany), 10 mM 4-(2-hydroxyethyl)-1-piperazineethanesulfonic acid (HEPES, Carl Roth, Karlsruhe, Germany) and penicillin (100 U/mL)/streptomycin (100 µg/mL) for 48 h. After this, the cells were incubated with BoNT-X (BoNT-A, BoNT-B and complex, BoNT-C complex: MiproLab, Göttingen, Germany, activity: >1.7 × 10^7^ MLD/mg) or Tetanus Toxin (TeT) (Sigma-Aldrich, Deisenhofen, Germany) in differentiation medium for 48 h. Pharmaceutical grade BoNT-A was provided by Croma-Pharma (Leobendorf, Austria) (Pharm 1) or bought from Allergan (Pharm 2) (Frankfurt am Main, Germany). Subsequently to the incubation with the toxin, the cells were pre-incubated with 100 µL of fresh medium for 10 min at 37 °C. The medium was aspirated and GLuc release was stimulated with 100 µL/well control (20 mM Hepes, pH 7.4, 136 mM NaCl, 4.7 mM KCl, 1.25 mM CaCl_2_ and 1.25 mM MgSO_4_, Carl Roth, Karlsruhe, Germany) or depolarization-buffer (20 mM Hepes, pH 7.4, 40.7 mM NaCl, 100 mM KCl, 1.25 mM CaCl_2_ and 1.25 mM MgSO_4_, Carl Roth, Karlsruhe, Germany) for 3 min at 37 °C. The supernatant was transferred into reaction vials and centrifuged at 100× *g* for 3 min to remove detached cells. To determine GLuc activity, 20 µL of the supernatant was mixed with 100 µL luciferase substrate solution and the luminescence was measured using Fluostar Optima (BMG Labtech, Offenburg, Germany). 

### 5.4. Western Blot Analysis

SIMA hPOMC1-26-GLuc cells (2 × 10^6^ cells/lysate) were lyzed in the Lämmli sample buffer (80 mM Tris/HCl at pH of 6.8), consisting of 2% (*w*/*v*) SDS, 5% (*w*/*v*) glycerol, 0.025% (*w*/*v*) bromphenol blue and 5% (*v*/*v*) 2-mercatoethanol (Carl Roth, Karlsruhe, Germany), which was homogenized by sonication. Insoluble material was removed by centrifugation (10,000× *g*, 15 min, 4 °C). Proteins were resolved by SDS-PAGE and transferred to a polyvinylidene difluoride (PVDF) membrane (Carl Roth, Karlsruhe, Germany). Membranes were blocked in 5% non-fat dry milk in 20 mM Tris, 136 mM NaCl and 0.1% (*v*/*v*) TWEEN 20 (Polyoxyethylenesorbitan monolaurate, TBS/Tween, Carl Roth, Karlsruhe, Germany) for 1 h at room temperature. Membranes were incubated with the first antibody (SNAP25 antibody, Cell signaling: 1:1000; all other antibodies: Santa Cruz 1:500) in TBS/Tween, containing 5% bovine serum albumin (Carl Roth, Karlsruhe, Germany), overnight at 4 °C and a horseradish-peroxidase-conjugated anti-rabbit or anti-mouse IgG (BIO-RAD, Munich, Germany) for 2 h at room temperature. The visualization of immune complexes was performed using chemoluminescence reagent Clarity Western ECL (BIO-RAD, Munic, Germany).

### 5.5. Real Time RT-PCR

The total RNA from differentiated SIMA hPOMC1-26-GLuc cells was isolated using peqGold Total RNA Kit (Peqlab, Darmstadt, Germany). A total of 1–2 µg total RNA were reverse-transcribed into cDNA using oligo dT as a primer and a M-MuLV Reverse Transcriptase (Thermo Scientific, Darmstadt, Germany). Hot start real-time PCR for the quantification of each transcript was carried out using 2 × Maxima SybrGreen qPCR mix (Thermo Scientific, Darmstadt, Germany) that consisted of 0.25 µM of each primer and 2.5 µL–5 µL of cDNA, which was diluted 1:10. PCR was performed with an initial enzyme activation step at 95 °C for 10 min, which was followed by 42 cycles of denaturation at 95 °C for 30 s, annealing at 57 °C for 30 s and extension at 72 °C for 1 min in a real-time DNA thermal cycler (CFX96™, 10 µL reaction volume, BIO-RAD; Munich, Germany). The oligonucleotides used are listed in Table 1. The expression level of BoNT substrates and receptors were calculated relative to GAPDH as a reference gene.

## Figures and Tables

**Table 1:** Oligonucleotide primers used for real-time qPCR.

Gene	Forward	Reverse
GAPDH	5′-TGATGACATCAAGAAGGTGG	5′-TTACTCCTTGGAGGCCATGT
SNAP25	5′-ACCAGTTGGCTGATGAGTCG	5′-GTTCGTCCACTACACGAGCA
VAMP-2	5′-CCATAGAGGGAGGGTGTTGC	5′-GTCCCCACCCTTACCTTGAG
SV2A	5′-GAAGGTGGTGCATCCAGTGA	5′-AGGCCTAGCATGCCTTTGTT
SytI	5′-TCCTGACCTGCTGCTTTTGT	5′-GGGTTTTGCCACCCAATTCC
SytII	5′-CATTGGACCCGTGGACAACT	5′-AGAACGCCCACAGTAAGCTG

Accession numbers for the genes were: GAPDH (AB062273), SNAP25 (NM_003081.4), VAMP-2 (AF135372.1), SV2A (NM_014849.4), SytI (NM_005639.2) and SytII (NM_177402.4).
